# Human Organoid and Supporting Technologies for Cancer and Toxicological Research

**DOI:** 10.3389/fgene.2021.759366

**Published:** 2021-10-22

**Authors:** Keisuke Sekine

**Affiliations:** Laboratory of Cancer Cell Systems, National Cancer Center Research Institute, Tokyo, Japan

**Keywords:** organoid, human cell, CRISPR (clustered regularly interspaced short palindromic repeat)/Cas9 (CRISPR associated protein 9)-mediated genome editing, iPS (induced pluripotent stem) cell, pancreatic cancer, toxicology

## Abstract

Recent progress in the field of organoid-based cell culture systems has enabled the use of patient-derived cells in conditions that resemble those in cancer tissue, which are better than two-dimensional (2D) cultured cell lines. In particular, organoids allow human cancer cells to be handled in conditions that resemble those in cancer tissue, resulting in more efficient establishment of cells compared with 2D cultured cell lines, thus enabling the use of multiple patient-derived cells with cells from different genetic background, in keeping with the heterogeneity of the cells. One of the most valuable points of using organoids is that human cells from either healthy or cancerous tissue can be used. Using genome editing technology such as clustered regularly interspaced short palindromic repeats (CRISPR)/CRISPR-associated protein, organoid genomes can be modified to, for example, cancer-prone genomes. The normal, cancer, or genome-modified organoids can be used to evaluate whether chemicals have genotoxic or non-genotoxic carcinogenic activity by evaluating the cancer incidence, cancer progression, and cancer metastasis. In this review, the organoid technology and the accompanying technologies were summarized and the advantages of organoid-based toxicology and its application to pancreatic cancer study were discussed.

## Introduction

**FIGURE 1:**
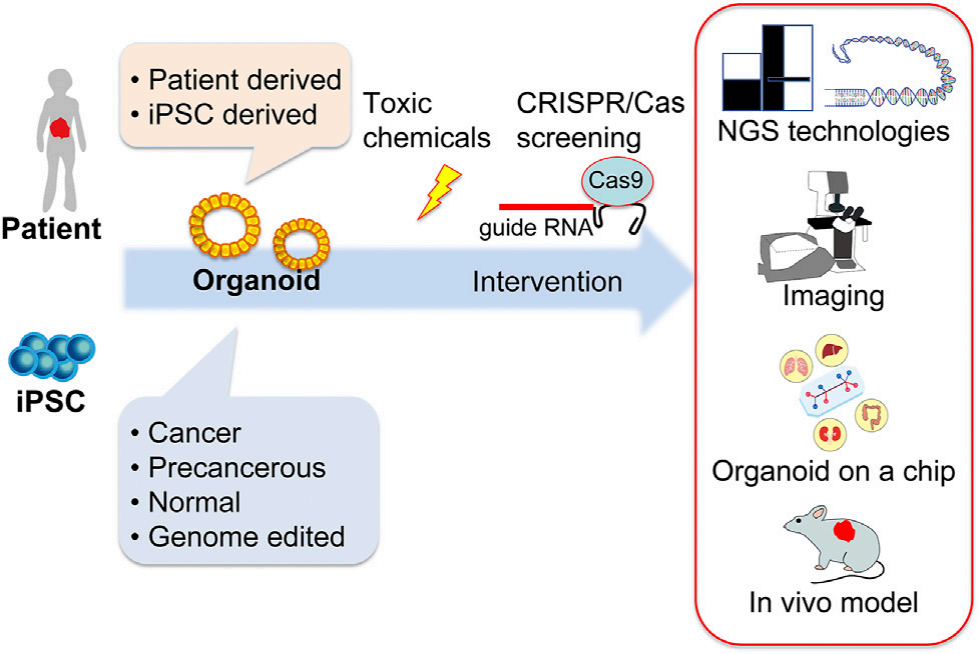
Schematic illustration of human organoid and supporting technologies for cancer and toxicological research. Human organoids can be established from patient- or human-induced pluripotent stem cells that are either cancerous, precancerous, normal, or genome-edited. These organoids serve as screening tools for toxic chemicals in combination with CRISPR/Cas technologies. Cutting-edge techniques, such as genome sequencing using NGS and RNA sequencing technologies, imaging technologies, culture devices, and the use of immunodeficient animal models for *in vivo* experiments support organoid-based human biology research. CRISPR/Cas, clustered regularly interspaced short palindromic repeats/CRISPR-associated protein; gRNA, guide RNA; iPSC, induced pluripotent stem cell; NGS, next-generation sequencing.

Carcinogenesis modeling using chemicals has been used to study cancer biology. Recently, studies using chemical carcinogenesis models have shifted to use genetically engineered mouse models (GEMMs), and the use of chemical carcinogenesis models has decreased. The exploration of cancer risk is important for society and to know each individual’s cancer risk, which can lead to early detection or even prevent cancer. It is now known that there are genetic and non-genetic risks for cancer. Non-genetic risks (i.e., environmental risks) include environmental radiation and chemicals, and we encounter these environmental non-genetic risks daily in the atmosphere and in food and drink. Among the non-genetic risks, exposure to toxic chemicals can be avoided if there is proper knowledge regarding these risks. In the past, as well as maybe at present, toxic chemicals have been used without knowledge of their toxicity and the resultant outcomes. The evaluation of such toxicity is essential to guarantee the safety of what we are exposed to. In addition, sophisticated models, such as GEMMs, are too simple and have included only a few mutations, however human cancer patients have several mutations and variations in mutations, most of which are still not well elucidated. GEMMs also have discrepancies regarding tumor onset, because most GEMMs have genetic mutations in all, or at least many, of the cells in the tissue, in contrast to human cancer or chemically induced carcinogenesis, in which a small fraction of cells acquires a precancerous mutation and, subsequently, the accumulation of mutations in these cells grants cancer onset. Therefore, detailed analysis of chemically induced carcinogenesis is still needed to elucidate the nature of cancer. Additionally, it is important to elucidate the characteristics of chemicals to induce and/or promote carcinogenesis. Some chemicals may not only induce cancer but can also accelerate progression or metastasis through additional mutation or epigenetic alteration. Toxicity has been evaluated using animals such as mice, rats, rabbits, and dogs, and for drugs, primates have also been used. However, the use of animals is time-consuming and labor-intensive. High-dose administration of chemicals to animals may sometimes affect animal health, which necessitates the humane sacrifice of animals, thus highlighting the need for an alternative to animal use, such as cell-based assays, for evaluating chemical carcinogenesis. Recent progress on organoid-based cell culture systems has enabled us to use patient-derived cells in conditions that better resemble cancer tissue compared to 2D cultured cell lines. In particular, organoids allow the handling of human cancer cells in conditions that resemble those in cancer tissue, and cells can be established more efficiently than in 2D cultured cell lines, which enable the use of multiple patient-derived cells with cells from different genetic backgrounds, in keeping with the heterogeneity of the cells. A remarkable benefit of using organoids is that human cells from either healthy or cancerous tissue can be used. Furthermore, genome editing technology, such as clustered regularly interspaced short palindromic repeats (CRISPR)/CRISPR-associated protein (Cas), can modify organoid genomes to, for instance, cancer-prone genomes. Normal, cancer, or genome-modified organoids can be used for evaluating whether specific chemicals possess genotoxic or non-genotoxic carcinogenic activity by assessing the rate of cancer incidence, progression, and metastasis. This review summarizes human organoid and supporting technologies (Figure 1). The benefits of organoid-based toxicology and its application to studies of pancreatic cancer are also discussed.

### Organoids

Organoid culture is similar to organ culture in developmental biology studies, but can be cultured from single cells isolated from the epithelial tissues of various species. Organoids can be also established from cancer tissue or induced pluripotent stem cell (iPSC)-derived epithelial cells. The main advantage of organoid technology is that human-derived cells can be more easily handled compared to classical 2D culture (Sato et al., 2009; Belair et al., 2018; Lou and Leung, 2018; Augustyniak et al., 2019; Sakib et al., 2019). In addition, human iPSC technology provides various human tissue-derived cells, which are almost impossible to obtain from human bodies; thus, iPSC technology allows us to explore human biology (Takahashi and Yamanaka, 2006; Takahashi and Yamanaka, 2016; Takahashi et al., 2007; Takebe et al., 2013; Camp et al., 2017).

### Organoids From Human Tissue (Patient-Derived Organoids)

Organoids can be established from healthy or diseased human tissue obtained by surgical resection, biopsy, autopsy, or abortion. Organoids can be used to compare and analyze normal and diseased states (Huch et al., 2013; Boj et al., 2015; Broutier et al., 2017). Patient-derived organoids are often compared with patient-derived xenograft (PDX) models. Both are established from patient specimens and demonstrate the characteristics of the tissue of origin, such as genomic and transcriptomic characteristics, and they can also potentially maintain the phenotypic responses of the primary tumor. Although they are similar, both have benefits and drawbacks. PDXs are established by directly transplanting the cancer tissue into immunodeficient mice, which enables the evaluation of drugs using cancer with human stromal cells that are originally present in tumors, although this stroma is gradually replaced by the host stromal cells with an increase in the number of xenograft passages (Delitto et al., 2015; Ben-David et al., 2017; Invrea et al., 2020). *In vitro* systems can be used to screen for drugs and to elucidate the mechanism of cancer survival and drug resistance. Patient-derived cancer organoids allow for chemical screening and genetic screening in human cells, which can lead to the identification of the genes involved in cancer cell survival and progression. Organoids derived from cancer tissue can also be transplanted into immunodeficient mice to form xenograft tumors that resemble the original tumor. Organoid-derived tumors are superior to PDX models because organoids can be modified before transplantation, such as by gene perturbation, gene overexpression, gene modification, or fluorescent protein labeling (Hidalgo et al., 2014; Aparicio et al., 2015; Bleijs et al., 2019; Koga and Ochiai, 2019). Organoid culture has evolved to mimic cancer tissues more precisely, including co-culture with stromal cells, such as fibroblasts or immune cells (Pape et al., 2021).

### iPSC-Derived Organoids

iPSC technology has enabled us to obtain and study human cells. Theoretically, researchers can obtain any type of human cell at any developmental stage. Human iPSC technology is mainly used for regenerative medicine and drug screening (Aurora and Spence, 2016; Sekine et al., 2020; Marsee et al., 2021; Yasui et al., 2021). From a cancer biology perspective, human iPSC technology is suitable for obtaining normal, healthy cells in order to study tumor onset (Kim et al., 2013; Sun et al., 2019; Frum and Spence, 2021). These normal cells, either from human iPSCs or tissue, can be used to evaluate tumor-inducing chemotoxicity. Because human iPSCs can be propagated infinitely, iPSC technology makes it possible to obtain a large number of cells with certain qualities. This characteristic also makes single-cell cloning for genome editing easy. In addition, human iPSC-derived cells can serve as a stromal cell population to mimic cancer-stromal cell interaction.

### Genome Editing With CRISPR/Cas9

CRISPR/Cas technology has provided us with tools to modify arbitrary nucleotides in the genome (Jinek et al., 2012). Cancer research has rapidly adopted the CRISPR/Cas technology because of the frequently reported genetic mutations in this field. Organoid models are amenable to the CRISPR/Cas technology. Moreover, low efficiency of genomic modification requires selection and cloning of modified cells in limited quantity; therefore, the characteristics of organoid technology pave the way to adopt and spread CRISPR/Cas technology in cancer research. The DNA endonuclease Cas has been found to give adaptive immunity against viruses and plasmids in bacteria and archaea. There are several CRISPR/Cas systems but the CRISPR/Cas9 system is the most widely used in mammalian cell genome editing. Within this system, Cas9 proteins are preloaded with small CRISPR RNAs (crRNAs) that act as guides for targeting a complementary target sequence, the protospacer, and the *trans*-activating crRNA (tracrRNA), which consists of stem-loop structure to bind to Cas9. The crRNA and tracrRNA are simplified as a chimeric single-guide RNA (sgRNA). A PAM (protospacer-adjacent motif) sequence is required immediately after the target DNA locus to be recognized by the Cas9-guideRNA ribonucleoprotein complex. The Cas9-guideRNA ribonucleoprotein complex induces a site-specific double strand break (DSB), which activates cellular DNA repair machinery. The CRISPR/Cas9 system is superior in terms of simplicity and cost- and time-efficiency over other nuclease platforms, such as transcription activator-like effector nucleases and zinc-finger nucleases. CRISPR/Cas9-based genome editing expresses its power in combination with organoid technology. The CRISPR/Cas9 system has several applications in cancer biology. One application of the CRISPR/Cas9 system is to knock out genes using the cellular mechanisms of DNA repair and non-homologous end joining (NHEJ). Knocking out tumor suppressor genes, such as TP53, in combination with toxic chemical administration might enhance the efficiency of screening. Arrays of guide RNAs to knockout genes is also used for screening important genes, and this approach can also be combined with toxic chemical administration to identify the oncogenic potential of chemicals in certain conditions (Agrotis and Ketteler, 2015). In any case, NHEJ usage of the CRISPR/Cas9 system only functions to knockout tumor suppressor genes if the researcher tries to enhance the tumorigenicity of the cells. Modification of the target gene is the ideal goal of genome editing. DSBs or single-strand nicks introduced by the Cas9-guideRNA ribonucleoprotein complex induce another DNA repair mechanism, known as homology-directed repair (HDR), which is suitable for genetic engineering because it introduces complementary DNA with desired modifications, such as activating mutations found in cancer patients. There are plenty of reviews on HDR-mediated nucleotide replacement, and many have detailed the use of genome editing and its utility for toxicological study in organoid technology (Drost et al., 2017; Roy et al., 2018; Fujii et al., 2019; Tang et al., 2019; Hendriks et al., 2020). HDR requires donor DNA with desired modifications in addition to the Cas9-guideRNA ribonucleoprotein complex. HDR takes place in order to repair the DNA damage introduced into the target gene by the Cas9-guideRNA ribonucleoprotein complex, and the donor DNA is used as a template to repair the damage. The desired mutation in the donor DNA is replaced and introduced into the genome of the target DNA. Although the probability of successful genome editing is still low, the CRISPR/Cas9 system using this method is enhanced compared to when it is used without double/single-strand breaks. The modifications introduce desired mutations, such as a certain oncogenic mutation, or fix mutations in the genome. It is even possible to fabricate cancer cells from normal cells by introducing multiple driver mutations. It is also useful to validate a mutation found in chemical screening by fixing it to check whether the mutation is responsible for the observed phenotype. The CRISPR/Cas9 system has several other useful applications, one of which is CRISPR activation (CRISPRa), in which modified Cas enzymes without endonuclease activity are fused to transcriptional activators such as VP64. The CRISPRa complex is recruited to the target promoter sequence, defined by gRNA-activated promoters and the endogenous gene expression of the target gene. CRISPR interference (CRISPRi) interferes with gene expression by fusing Cas proteins with or without inhibitors such as Krüppel-associated box proteins, and is therefore the opposite technology to CRISPRa. Both technologies are useful to precisely modify gene expression. Thus, CRISPR/Cas-based technologies extend our options to explore toxicology and cancer biology using organoids.

### Other Useful Technologies

With the evolution of next-generation sequencing (NGS) technology and other technologies, exhaustive evaluation methods can be utilized in cancer fields. Whole-exome and whole-genome sequencing has changed the field of cancer research (Goodwin et al., 2016; Mora-Castilla et al., 2016; Kamps et al., 2017; McCombie et al., 2019) and has made it easy to find mutations after toxic chemical administration. In addition, we can now investigate whole transcriptomes in cancer tissues and cells at the single-cell level. Single-cell RNA sequencing technology is especially suitable to patient-derived organoids because it preserves the heterogeneity of the cells (Camp and Treutlein, 2017; Camp et al., 2018). Single-cell level analysis spans not only transcriptome sequencing but also whole-genome, proteome, and metabolome analysis (Irish et al., 2006; Restrepo-Pérez et al., 2018; Das et al., 2020; Xing et al., 2020; Specht et al., 2021). These single-cell level multi-omics analyses have good compatibility with organoid technology.

The other important technology relevant to human organoid technology is the development of immunodeficient animals. Severely immunodeficient animals are required to develop human cell transplantation models because of the increased availability of human samples. In addition to mice such as NOD/scid/IL-2Rγ-null mice (NOG/NSG), immunodeficient rats, and even immunodeficient apes have been developed for transplantation, and human organoid technology can utilize this technology (Ito et al., 2002; Shultz et al., 2007; Mashimo et al., 2012; Sasaki et al., 2016). Namely, organoid technology evolves together with these cutting-edge technologies.

### Pancreatic Cancer

This review focuses on pancreatic cancer as a model for the use of organoid technology in cancer and toxicological research because of the substandard treatment options currently available and the need to develop better treatment alternatives. Pancreatic cancer has one of the worst prognoses, and the five-year survival rate is around 9% (Vincent et al., 2011; Dreyer et al., 2017; Thompson et al., 2020). It is speculated that pancreatic cancer will become the second highest cause of cancer mortality by 2030 in the US (Siegel et al., 2017). Variations in the mutations in pancreatic cancer are relatively low, and the major driver mutations are known as “the big four,” namely, KRAS, TP53, CDKN2A, SMAD4, which are present in most (50–95%) pancreatic cancer patients. Even though driver mutations are restricted, many other genes are mutated in pancreatic cancer and are known to be related to the prognosis. To find druggable mutations, patient stratification is necessary to improve patient prognosis (Singhi et al., 2019; Pishvaian et al., 2020); however, there are currently no good strategies to achieve this goal. In this situation, toxic chemical oncogenesis screening using GEMMs might be a good strategy to find mutations which drive or repress cancer progression. The GEMMs with the KRAS mutation should be precancerous or have low aggressiveness and is suitable to assess additional mutations introduced by toxic agents that promote cancer onset and aggressiveness such as metastasis (Long et al., 2021). Once mutations related to cancer onset, dissemination, and metastasis are identified, the gene and its peripheral signal molecules might serve as a druggable targets to repress cancer. Although this looks quite possible, the additional mutations found in GEMMs with the KRAS mutation at tumor onset are known to be unrelated to the mutations found in human pancreatic cancer. This leads to the importance of using human patient-derived cells. PDXs and patient-derived cancer organoids are suitable for screening toxic agent-induced mutations (Boj et al., 2015; Seino et al., 2018; Huang et al., 2020). PDXs and cancer organoids without metastatic potential can be used to screen metastatic mutations and mutations that strongly enhance tumor aggressiveness induced by toxins; however, to assess the mutations that contribute to cancer development, malignant PDXs and cancer organoids are not suitable. Non-cancer normal cells obtained as an accessary tissue at the time of cancer resection do not establish tissue mass even if transplanted into immunodeficient mice. Organoid technology is the only way to utilize non-cancer normal cells. Normal cells can be maintained in 3D culture, and driver mutations such as the KRAS mutation can be introduced by CRISPR/Cas9-based genome editing. In addition to the KRAS mutation, one or more of the “big four” mutations may be introduced in a context-dependent manner. These cells, with a few fundamental mutations, are not obtained from cancer tissue because cancer cells already have many other passenger mutations. The cells with few mutations might not produce tumors when transplanted to immunodeficient mice. This is assumed from the evidence that GEMMs that have mutations from embryonic pancreas development do not develop cancer at birth, suggesting that additional genetic and/or epigenetic mutation(s) are required to develop cancer. Therefore, cells with few mutations are suitable for screening to find mutations that are important for pancreatic tumorigenesis by treating them with toxins that introduce additional mutations to induce tumorigenesis. Throughput is also important for screening, and organoids are easier to handle in large volumes compared to mice. As the first step in screening, organoids treated with individual toxins, which may be different toxins, in individual culture can be transplanted together into the same mouse. If cancer emerges, the treated cells can be transplanted individually or as a small group as the second screening step. Whole-exome or whole-genome analysis will help to find the mutations responsible for the observations. However, while searching for meaningful mutations looks promising, our efforts may be wasted. It is true that mutations in pancreatic cancer are restricted to small set of genes, and there is the possibility that there are no additional key mutations. Therefore, after transplantation of a toxin-treated organoid, a resultant tumor may emerge due to epigenetic modification. Analysis of the gene expression and epigenetic status will help to understand pancreatic cancer development as well as the hidden influences of the toxins on epigenetic.

### Limitations and Future Research

So far, the advantages of using organoids and the peripheral technology in cancer toxicology were discussed. However, there are limitations and issues that need to be solved in the future. Not only for toxicology, but in *in vitro* drug/chemical evaluation, drugs and chemicals are not metabolized in metabolic organs such as the intestines and/or liver, and also the effect in the whole body cannot be evaluated. In this context, researchers are developing the possibility of multi-organ evaluation using a system such as organs on a chip with multiple organs, in which several types of tissue-derived cells or organoids derived from pluripotent stem cells are assembled in a multi-compartment or microfluidic chamber to communicate *via* fluid to mimic the circulatory system (Khetani and Bhatia, 2008; Lee et al., 2013; Skardal et al., 2016; May et al., 2017; Takebe et al., 2017). This sounds promising, but even if it was accomplished, discrepancies will remain between this system and the *in vivo* situation, and it may not be suitable for high-throughput screening. Regarding the throughput of organoid technology, unlike 2D cultured cells, the low throughput of 3D cultured cells might hinder the screening of chemicals. Advances in the field of robotics may help to improve the handling of thousands of chemicals; therefore, we need to await the maturation of robotic technologies in cell handling (Louey et al., 2021; Ostrop et al., 2021; Yasui et al., 2021). The other limitation is the deficiency of an immune system in the *in vitro* system. Several studies have involved the introduction of immune cells in *in vitro* culture systems, but a full response of human immunity is not reproducible. If the human organoids are transplanted to immunodeficient mice, the hematopoietic system would need to be humanized if an immune system is required. Nevertheless, as in many other situations, there is no absolute experimental systems, and several experimental systems should be combined to obtain reliable results.

## Summary

Organoid technology in combination with CRISPR/Cas-based genome editing technology, NGS, and other cutting-edge technologies presents great promise in the advance of our knowledge into toxicology. In particular, organoids allow us to handle human cancer cells in conditions that more closely resemble cancer tissue compared to 2D cultured cell lines. In addition, cells can be established more efficiently in organoids than in 2D cultured cell lines, enabling the use of multiple patient-derived cells with different genetic background, which sometimes influences the reactions to chemicals. Furthermore, organoids can be handled in large quantities, their DNA sequences can be modified (disrupted and corrected) using genome editing technology, and they can be analyzed using other cellular and molecular dissection technologies. We need to explore how chemicals influence our health. We believe that we have enough knowledge to exclude unhealthy, toxic chemicals but it is still unknown why cancer incidence is increasing and whether this is due to the increase life expectancy. The increase of cancer incidence of all cancers might be due to the fact that there are many potentially harmful substances in our food and/or environment. It will be important to establish a systematic evaluation strategy and evaluation criteria for toxic agents using organoids.
